# Next‐generation sequencing identifies novel *CACNA1A* gene mutations in episodic ataxia type 2

**DOI:** 10.1002/mgg3.196

**Published:** 2016-01-20

**Authors:** Neven Maksemous, Bishakha Roy, Robert A. Smith, Lyn R. Griffiths

**Affiliations:** ^1^ Genomics Research CentreInstitute of Health and Biomedical Innovation (IHBI)Queensland University of Technology (QUT)Q Block 60 Musk Ave Kelvin Grove CampusBrisbaneQueenslandAustralia

**Keywords:** AmpliSeq custom panel, *CACNA1A*, episodic ataxia type 2, next‐generation sequencing

## Abstract

Episodic Ataxia type 2 (EA2) is a rare autosomal dominantly inherited neurological disorder characterized by recurrent disabling imbalance, vertigo, and episodes of ataxia lasting minutes to hours. EA2 is caused most often by loss of function mutations of the calcium channel gene *CACNA1A*. In addition to EA2, mutations in *CACNA1A* are responsible for two other allelic disorders: familial hemiplegic migraine type 1 (FHM1) and spinocerebellar ataxia type 6 (SCA6). Herein, we have utilized next‐generation sequencing (NGS) to screen the coding sequence, exon‐intron boundaries, and Untranslated Regions (UTRs) of five genes where mutation is known to produce symptoms related to EA2, including *CACNA1A*. We performed this screening in a group of 31 unrelated patients with EA2 symptoms. Both novel and known mutations were detected through NGS technology, and confirmed through Sanger sequencing. Genetic testing showed in total 15 mutation bearing patients (48%), of which nine were novel mutations (6 missense and 3 small frameshift deletion mutations) and six known mutations (4 missense and 2 nonsense).These results demonstrate the efficiency of our NGS‐panel for detecting known and novel mutations for EA2 in the *CACNA1A* gene, also identifying a novel missense mutation in *ATP1A2* which is not a normal target for EA2 screening.

## Introduction

Episodic Ataxia type 2 (EA2) is an autosomal dominantly inherited paroxysmal cerebral disorder that demonstrates variable expressivity and starts in childhood or early adolescence (age range 2–32 years). EA2 is characterized by episodes of ataxia, vertigo, and nausea lasting minutes to hours. The frequency of attacks ranges from once a year to four times a week. Attacks can be triggered by stress, exertion, caffeine, alcohol, fever, and heat. Acetazolamide (Griggs et al. 1978) and 4‐aminopyridine (Strupp et al. 2004) have been shown to be effective in treating EA2, particularly in reducing attack frequency and severity.

The calcium channel gene (*CACNA1A)* gene (MIM: 601011) which covers 300 Kb with 47 exons, is located at chromosome 19p13 (Kramer et al. 1995; Teh et al. 1995) and is the only gene in which mutations are known to cause EA2 (Ophoff et al. 1996). The gene codes for the *α*1A pore‐forming subunit of Ca^2+^ voltage‐gated Cav2.1 channels and is widely expressed throughout the central nervous system (CNS) (Mori et al. 1991; Westenbroek et al. 1995). It is involved in a variety of Ca^2+^‐dependent processes, including mediating the entry of Ca^2+^ ions into excitable cells, muscle contraction, hormone or neurotransmitter release, and gene expression (Tsien et al. 1991).

Mutations in the *CACNA1A* gene have been found to be responsible for three disorders with autosomal dominant inheritance: EA2 (MIM: 108500), familial hemiplegic migraine type 1 (FHM1; MIM: 141500), and spinocerebellar ataxia type 6 (SCA6; MIM: 183086). Clinical overlap between the three disorders in terms of symptoms has been previously reported (Jodice et al. 1997; Mantuano et al. 2003; Romaniello et al. 2010). Nonsense mutations (Ophoff et al. 1996), deletions (Riant et al. 2008; Labrum et al. 2009), and missense mutations in *CACNA1A* gene have all been found to lead to loss‐of‐function of recombinant human CaV2.1 channels in heterologous expression systems found to cause EA2.

Since the 1970s, most DNA diagnosis is undertaken by “gold standard” traditional DNA Sanger sequencing. This is an accurate but slow and expensive means of diagnosis. Additionally, in order to avoid extensive costs to patients or health systems, the practice typically involves screening only regions where mutations are known, or more likely, to occur, limiting the ability of testing to identify unusual mutations causing disease. Those who fail initial screenings must thus go through additional rounds of testing. Therefore, testing for EA2 using Sanger sequencing is difficult, time consuming, and expensive due to the number and size of sequences investigated. In contrast, next‐generation sequencing (NGS) approaches have opened the door for massive parallel sequencing of targeted genes, as well as whole‐exome and whole‐genome sequencing. The AmpliSeq custom panel technique used here is an appropriate method to screen mutations in genes which cause clinically overlapping disorders.

We have developed an AmpliSeq custom panel comprising the genes most related to episodic ataxia type2 and FHM in terms of causation of symptoms (*CACNA1A, ATP1A2, SCN1A, NOTCH3,* and *KCNK18*) in order to improve mutation detection. We screened a cohort of 31 unrelated patients with clinical diagnosis of EA2 for whom primary Sanger sequencing had failed to find a causative mutation using the Ion Torrent NGS platform.

## Materials and Methods

### Patients

The Genomics Research Centre (GRC) diagnostics clinic began diagnostic testing in 1999 and to date has tested approximately 1050 patients for (EA2), (FHM) and cerebral autosomal dominant arteriopathy with subcortical infarcts and leukoencephalopathy (CADASIL). In the early stages of the clinic's operation, causative mutations were identified using the traditional Sanger sequencing approach. During this period, DNA testing was undertaken in 167 EA2 patients according to the request of certified clinical neurologists around Australia and New Zealand. Informed consent was obtained by physicians for all patients for genetic testing and this study was approved by the Queensland University of Technology (QUT) Ethics Committee. Of the 167 patients that had been screened previously for mutations in selected exons in *CACNA1A* gene, few were found to have genetic aberrations. This prompted the development of the NGS test. From our 167 patients, 31 index cases underwent advanced testing, due to the presence of clinical EA symptoms without detection of genetic mutations by primary Sanger sequencing.

### Migraine panel characteristics and ion torrent sequencing

In the development of the panel, we chose five genes (Table 1) that were known to be involved in monogenic neurological disorders, including EA2. The ampliseq.com web designer was utilized to design primer pools to our specified genomic regions, according a reference human genome (hg 19 for our study). The primers for this panel of genes cover exonic regions, exon‐intron boundaries as well as 3′ and 5′ Untranslated Regions (UTRs). In total, this amounts to 34.9 kb of sequencing region, with the primers allowing for sequencing of 91.97% of our original target regions, using 274 primer pairs in two highly multiplexed reactions requiring only 10 ng/reaction of input DNA per sample.

**Table 1:** Migraine panel candidate genes

Neurological genes	Transcript	Chromosome	Gene length (Kb)	Number of exons	Targeted length (Kb)	Disease
CACNA1A	NM_001127221.1	Chr19p13	~300	47	~11	FHM1, EA2, SCA6
ATP1A2	NM_000702.3	Chr1q21‐23	~28	23	~5.5	FHM2
SCN1A	NM_006920.4	Chr2q24.3	~160	26	~9.2	FHM3, Epilepsy
NOTCH3	NM_000435.2	Chr19p13.2‐13.1	~41	33	~8.1	CADASIL
KCNK18 (TRESK)	NM_181840.1	Chr10q25.3	~13	3	~1.1	Familial migraine

In brief, sequencing of the amplified regions for the 31 genomic DNA samples were then conducted using the Ion AmpliSeq library kit (Catalog Number 4480441 Rev A.0; Life Technologies Mulgrave, Victoria, Australia), followed by template preparation using the Ion OneTouch instrument (Catalog Number 4480974 Rev 5.0; Life Technologies), and finally were sequenced on the Ion Torrent Personal Genome Machine (PGM) using the sequencing 200 v2 kit protocols (Catalog Number 4482006 Rev 3.0; Life Technologies). One of 16 unique barcodes (Ion Xpress) was used to provide identification for each sample on a particular sequencing reaction. Library quality and quantity was assessed using the Agilent DNA High Sensitivity Bioanalyzer Kit (Agilent Technologies, Santa Clara, CA, USA). Two Ion 316 v2 Chips were used for sequencing allowing multiplexing of the 16 barcoded samples. The number of samples used per chip provided scope for around 100X read depth of coverage at each base, allowing accurate detection of mutations.

### NGS sequencing analysis

Data from the PGM runs were processed initially using the Ion Torrent platform‐specific pipeline software (Torrent Suite v4.0.2 (Life Technologies, Mulgrave, Victoria, Australia)) to generate sequence reads, trim adapter sequences, filter, and remove lower quality reads according to the designed bed file provided by AmpliSeq designer. Generated sequence files were aligned to the human complete genome (hg19). DNA and protein sequences from NGS and later Sanger sequencing were compared with the NCBI reference sequences (Pruitt et al. 2012), and the UCSC genome browser (Dreszer et al. 2012). All rs ID numbers, locations, allele frequencies and genotypes for known variants were determined based on single‐nucleotide polymorphisms (SNPs) reported in dbSNP database (Sherry et al. 2001) and further verified in the 1000 Genomes datasets. All variants detected were visually confirmed using the Integrative Genome Viewer (IGV2.3) software (Thorvaldsdottir et al. 2013) and Ion Reporter Software (Life Technologies) were used to annotate the variants.

All potentially causative mutations were further investigated by conventional Sanger Sequencing using standard protocols (Roy et al. 2012). Forward and reverse primer sequences for the validations are listed in (Table 2). PCR products were used as templates for sequencing with BigDye Terminator reagents on a 3500 DNA sequencing Analyzer (Life Technologies). Sanger sequence traces were aligned to the gene‐specific reference sequence (NCBI build) using BLAST and CLUSTAL W, and visualized for manual verification of mutations and variants with Chromas 2.33 software (Technelysium, Brisbane, Queensland, Australia).

**Table 2:** List of primers used for PCR amplification of exons with novel *CACNA1A* mutations and variants detected by NGS

Exon	Sense primer (5′‐3′)	Antisense primer(5′‐3′)
3	acg ctg acc ttg cct tct ct	caa cca aaa gcc tcg taa tc
6	tcc ctt ccc ttt tgt aga tg	gtg ggg ctg tgt tgt cct t
7	gac aga gcc aca aga gaa cc	agc aaa gag gag tga gtg gg
8	ata ctc tgg ctt ttc tat gc	gca tga ctc tct ttg tac tc
9	gca gag aat ggg ggt gg	ctg agg tgg gtt tag agc ag
12	caa gcc taa cct cct ctc tg	tca ttc cag gca aga gct g
13	att tgg agg gag gag ttt gg	tca ctt tcc caa ctt tct gg
14	cag aaa gtt ggg aaa gta gc	ttg aat tcc tgt gaa gga c
27	ctg ctt ccc aag cag tct ag	tcc tgg ata gat ttc cag tc
34	aga agc cac tgg agg aat ggc	att atc aga gca ggt ccc ctt c
37	tgt gaa ccc att gcc tgc a	tgg gaa tga ctg cgc ttg c

To predict the damaging effect of non‐synonymous single‐nucleotide substitutions on protein structure, function, or phenotype, we used the available online tools, such as SIFT (Ng and Henikoff 2001) (http://sift.jcvi.org/www/SIFT), Polyphen2 (Adzhubei et al. 2010) (http://genetics.bwh.harvard.edu/pph2/) and finally Mutation Taster (Schwarz et al. 2010) (http://www.mutationtaster.org/). Each software package was used to estimate the pathogenicity effect of every suspected variant, such as exonic nonsynonymous/synonymous, intronic, and variants in the 5′ and 3′ UTRs, though SIFT and Polyphen2 only estimate pathogenicity based on coding sequence changes.

Where patients were also family members seeking confirmation of mutation status, segregation analysis for the available family members was conducted to further determine the impact of novel mutations/variants with the phenotype.

## Results

### Sequencing output analysis

Genomic DNA from 31 patients with (EA2) phenotypes was sequenced using a NGS approach. Resultant Ion Torrent PGM sequence data were analyzed using the Ion Torrent platform‐specific software Torrent Suite v4.0.2 as detailed above. Sequencing via PGM generated an average sequencing of 3,136,750 total reads per Ion 316 Chip, 455 Mb total bases detected, and 450 Mb of final readable data with 99% of total bases aligned to the human complete genome (hg19) and an average amplicon size of 147 bp.

The complete summary of data for each detected functional variant, mutation, and amino acid neutral novel variant can be found in Table 3.

**Table 3:** Novel functional mutations and variants detected by NGS

Case ID	Locus	Family Histroy	Genotype	Ref	Type	Genes	Location	Exon	Transcript	Coding	Amino acid change	Variant effect	SIFT	Polyphen	Mutation taster	Coverage	Allele coverage	Reference
29+	chr19:13318233	YES	A/G	A	SNV	CACNA1A	utr_3	–	NM_023035.2	–	–	–	–	–	D	74	A = 39, G = 35	–
6	chr19:13320393	YES	G/T	G	SNV	CACNA1A	Intronic	−	NM_023035.2	–	–	–	–	–	T	276	G = 133, T = 143	–
15	chr19:13339582	–	G/C	G	SNV	CACNA1A	Exonic	37	NM_023035.3	c.5559C>G	p.Tyr1853*	Nonsense	D	D	D	400	G = 205, C = 195	Giffin et al. (2002)
9	chr19:13345741	–	A/G	A	SNV	CACNA1A	Exonic	34	NM_023035.2	c.5246T>C	p.Leu1749Pro	Missense	D	D	D	261	A = 138, G = 123	–
	chr19:13370426	–	C/T	C	SNV	CACNA1A	Exonic	27	NM_023035.2	c.4343G>A	p.Trp1448*	Nonsense	D	D	D	169	C = 93, T = 76	Jen et al. (2004)
8	chr19:13419049	YES	TGA/T	TGA	INDEL	CACNA1A	Exonic	14	NM_023035.2	c.1799_1800delTC	p.Leu600 fs*41	Frameshift deletion	–	–	D	395	TGA = 220, T = 175	–
30	chr19:13419266	YES	C/T	C	SNV	CACNA1A	Exonic	13	NM_023035.2	c.1748G>A	p.Arg583Gln	Missense	D	D	D	400	C = 205, T = 195	rs121908217
3	chr19:13419338	–	ATAACCTAG/ATAG	ATAACCTAG	INDEL	CACNA1A	Splicesite_5	13	NM_023035.2	c.1672‐1_1675deletionGGTTA	V558Sfs*13	Frameshift deletion	–	–	D	394	ATAACCTAG = 198, ATAG = 196	–
26	chr19:13423536	YES	C/T	C	SNV	CACNA1A	Exonic	12	NM_023035.2	c.1618G>A	p.Gly540Arg	Missense	D	D	D	400	C = 206, T = 194	Rajakulendran et al. (2010)
22	chr19:13423557	–	C/T	C	SNV	CACNA1A	Exonic	12	NM_023035.2	c.1597G>A	p.Glu533Lys	Missense	D	D	D	399	C = 187, T = 212	Scoggan et al. (2006)
27	chr19:13443707	–	C/A	C	SNV	CACNA1A	Exonic	9	NM_023035.2	c.1231G>T	p.Gly411Trp	Missense	D	D	T	399	C = 198, A = 201	–
24	chr19:13445231	YES	G/C	G	SNV	CACNA1A	Exonic	8	NM_023035.2	c.1159C>G	p.Arg387Gly	Missense	D	D	D	256	G = 127, C = 129	–
13+	chr19:13446718	YES	G/A	G	SNV	CACNA1A	Exonic	7	NM_023035.2	c.984C>T	p.(=)	Synonymous			D	400	G = 210, A = 190	–
17	chr19:13470466	–	ACAGT/A	ACAGT	INDEL	CACNA1A	Exonic	6	NM_023035.2	c.928_931delACTG	p.Thr310 fs*5	Frameshift deletion	D	D	D	397	ACAGT = 216, A = 181	–
13+	chr19:13470494	YES	C/T	C	SNV	CACNA1A	Exonic	6	NM_023035.2	c.904G>A	p.Asp302Asn	Missense	D	D	D	400	C = 205, T = 195	Burk et al. (2014) Jaffer F et al.
29+	chr19:13470563	YES	G/A	G	SNV	CACNA1A	Exonic	6	NM_023035.2	c.835C>T	p.Arg279Cys	Missense	D	D	D	400	G = 132, A = 268	–
28	chr19:13563744	–	C/A	C	SNV	CACNA1A	Exonic	3	NM_023035.2	c.485G>T	p.Gly162Val	Missense	D	D	D	399	C = 195, A = 204	–
19	chr19:15276625	–	G/A	G	SNV	NOTCH3	Exonic	30	NM_000435.2	c.5640C>T	p.(=)	Synonymous			D	104	G = 42, A = 62	–
18	chr1:160100269	–	C/T	C	SNV	ATP1A2	Exonic	13	NM_000702.3	c.1709C>T	p.Thr570Met	Missense	D	D	D	400	C = 210, T = 190	–
10	chr1:160111600	–	G/T	G	SNV	ATP1A2	utr_3	−	NM_000702.3	–	–	–	–	–	T	77	G = 44, T = 33	–

D, damaging; T, Tolerated. +, Multiple variants found in patient.

### Variants analysis

Our panel sequences five genes associated with different neurological disorders including EA2, which all have overlapping phenotypes as part of our comprehensive diagnostics tool.

After variant filtering, annotation, and interpretation, 127 different variants were identified among the 31 patients in the five sequenced genes with an average of 40 variants/patient. The bulk of these are unremarkable common SNPs and are not of clinical interest. In the following subsections, we will discuss the more unusual variants detected in these cases.

### Identification of known and novel mutations

Nucleotide changes resulting in changes in highly conserved amino acid residues and predicted to be pathogenic with at least two of the prediction tools used, were considered to be mutations.

In total, 15 of 31 (48.4%) of our patients carried probable disease‐causing nonsynonymous, nonsense, and small frameshift deletion mutations in the *CACANA1A* and *ATP1A2* genes (Table 3). As expected on the basis of the requested EA2 test, 14 of the 15 identified mutations were detected in the *CACNA1A* gene and only one was unexpectedly detected in *ATP1A2* gene.

**Figure 1:**
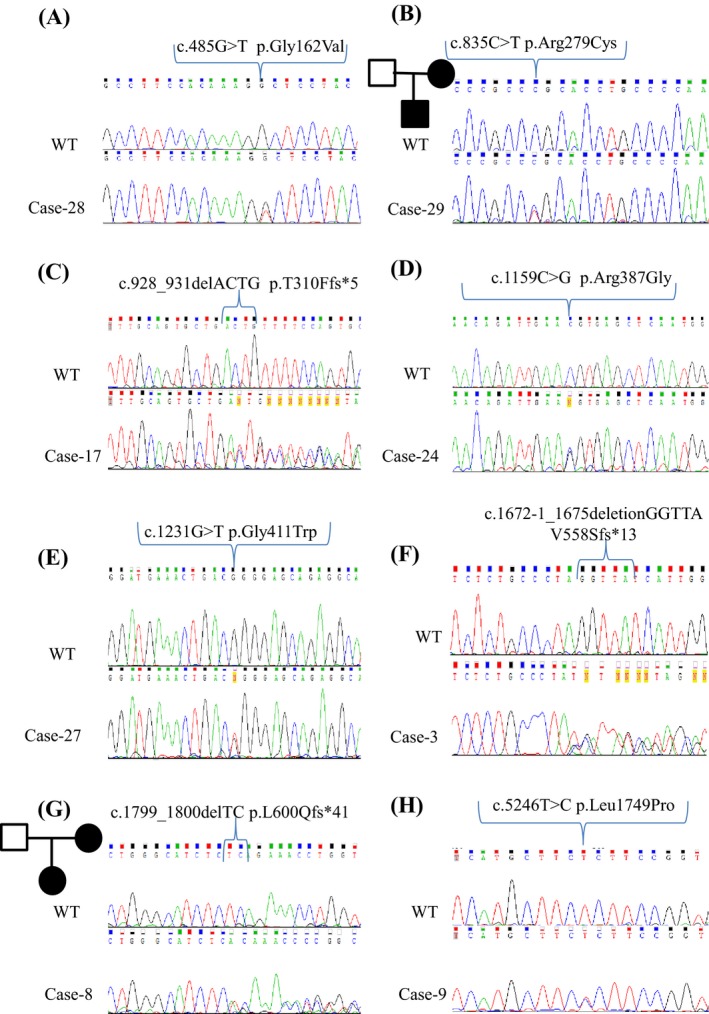
Sequences of the eight novel genetic variants in calcium channel gene *CACNA1A* identified by next‐generation sequencing. Five heterozygous exonic missense point mutations (A) in exon 3; (B) in exon 6; (D) in exon 8; (E) in exon 9; and (H) in exon 34. Three small frameshift deletion mutations (C) in exon 6; (F) in exon13; and (G) in exon 14.

**Figure 2:**
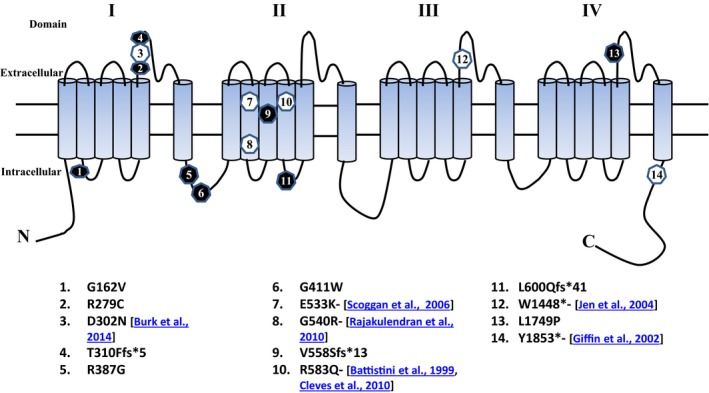
Mutations in the α1A subunit of the voltage‐gated Cav2.1 Ca^2+^ channel encoded by Episodic Ataxia type 2 (EA2) calcium channel gene CACNA1A. The protein is located in the plasma membrane and contains four repeated domains (I‐IV), each encompassing six transmembrane segments (S1‐6). ○, Known mutations, ●, Novel mutations. Numbers in the symbol correspond to the mutation listed in order.

Among the 14 detected mutations in the *CACNA1A* gene (Table 3, Figs. 1, 2), six were previously described disease‐causing mutations: two missense mutations in exon 12 p.Glu533Lys (Scoggan et al. 2006), p.Gly540Arg (Rajakulendran et al. 2010); two missense mutations in exon 6 and 13 (Cases 13, 30), p.Asp302Asn (Burk et al. 2014) p.Arg583Gln (rs121908217) (Battistini et al. 1999; Cleves et al. 2010) and; the two nonsense mutations found in exon 27 and 37, p.Trp1448Ter (Jen et al. 2004) and p.Tyr1853Ter (Giffin et al. 2002).

The remaining eight mutations in *CACNA1A* were previously undescribed and estimated by the *in silico* tools as pathogenic. In the patients tested, there were three novel frameshift deletions: Case 17, c.928_931delACTG p.Thr310 fs*5; Case 3, c.1672‐1_1675deletionGGTTA Val558Ser fs*13; and case 8, c.1799_1800delTC p.Leu600 fs*41 detected in exon 6, 13, and 14, respectively. Other than the frameshifts, there were five novel nonsynonymous mutations scattered throughout five different exons: in exon3 (Case 28) p.Gly162Val; in exon6 (Case 29) p.Arg279Cys; in exon8 (Case 24) p.Arg387Gly; in exon9 (Case 27) p.Gly411Trp; and in exon34 (Case 9) p.Leu1749Pro.

Interestingly, one novel mutation was identified in one of the 31 EA2 patients screened (Case 18) in exon13 of the *ATP1A2* gene (c.1709C>T, p.Thr570Met) and not the *CACNA1A* gene. Like the mutations detected in *CACNA1A* this *ATP1A2* mutation was predicted to be deleterious by the three prediction tools used here.

### Identification of novel variants

In addition to the novel and known mutations identified in these cases, there were a number of novel non amino acid changing variants identified. First, a synonymous novel variant in exon 7 c.984C>T found in the same patient (Case 13) who was carrying a known mutation (p.Asp302Asn) and both the synonymous and nonsynonymous variants were confirmed with Sanger in the index case and the available DNA of the only one family member also sent for testing (a sibling). Similarly Case 29, was found to have a novel missense mutation (p.Arg279Cys) in *CACNA1A* gene in exon 6 along with novel potentially damaging 3′UTR variant (Table 3).

Moreover, there were three unique variants identified in three different patients, the first, a synonymous variant was identified in Case 19 in the *NOTCH3* gene and was predicted to be damaging. The second novel variant was detected in case 6 in *CACNA1A*, it is a new sequence and was predicted to be tolerated, and finally, a new 3′UTR variant was detected (Case 10) in the *ATP1A2* gene, also predicted to be tolerated by Mutation Taster software.

### Identification of rare SNPs

In 10 of 16 cases for whom our panel could not detect known or unknown mutations/damaging variants, several rare SNPs were detected, which are listed in Table 4. All have variable pathogenic effect when analyzed by Mutation Taster software, most of them were unique to patients without any known or novel pathogenic genetic aberration identified in the five screened genes.

**Table 4:** Rare variants <0.1% detected in our 16/31 cases (Ref hg19)

Case ID	Locus.	Ref	Type	Gene	Location	Length	Coding	Amino acid change	Variant Effect	PhyloP	dbSNP	MAF
25	chr2:166845891	A	SNV	SCN1A	utr_3	1				0.63	rs150155252	0.003
31	chr2:166848003	G	SNV	SCN1A	Exon26	1	c.5749C>G	p.Arg1917Gly	Missense	1.44	rs121917956	0.001
21	chr2:166848367	C	SNV	SCN1A	exonic26	1	c.5385G>A	WT	Synonymous	2.73	rs140237315	0.008
23	chr2:166870199	G	SNV	SCN1A	Intron18	1				1.18	rs76220226	0.006
23	chr2:166870221	A	SNV	SCN1A	Intron18	1				−1.23	rs76743139	0.005
20	chr19:13317274	T	SNV	CACNA1A	utr_3	1				−0.2	rs145764460	0.001
3,4	chr19:13317758	G	SNV	CACNA1A	utr_3	1				1.74	rs370662140	−
11,18	chr19:13317825	T	SNV	CACNA1A	utr_3	1				0.18	rs111240372	0.006
7	chr19:13419235	G	SNV	CACNA1A	Exon13	1	c.1779C>G	WT	Synonymous	2.37	rs16012	0.008
10	chr19:13482464	G	SNV	CACNA1A	Intron4	1				0.16	rs202061083	0.007
11	chr19:13482554	C	SNV	CACNA1A	Exon4	1	c.579G>A	WT	Synonymous	−0.51	rs41276894	0.006
7	chr19:15271377	G	SNV	NOTCH3	utr_3	1				−0.09	rs117165744	0.006

### Mutation validation and segregation analysis

All known and novel mutations were further validated and confirmed with conventional Sanger sequencing (Fig. 1). In four of the 15 cases (Cases: 8, 13, 29, and 30), DNA samples from one of the patient's parents or siblings was also requested for genetic testing in addition to the index case. Upon detection of the mutation in the index cases, these family members were also tested to help confirm mutation pathogenicity. Segregation of the individual mutations with similar phenotype as the index case as noted in the testing request was confirmed within all four families (see schematic pedigree for novel mutations in Fig. 1).

In summary, the mutational spectrum for EA2 patients comprised six known and nine unknown mutations. Among them are three new small deletions (*CACNA1A* p.Thr310 fs [4 bp]; Val558Ser fs*13 [5 bp]; and p.Leu600 fs [2 bp]) and five new missense mutations all predicted to cause EA2.

### Clinical context

The clinical features varied among the 31 tested cases (18 males and 13 females). Frequency of attacks ranged from weekly to yearly attacks, and duration of attacks ranged from 30 min to hours, 12/31 had family history of a similar phenotype to the index case. Some cases had more extensive clinical symptoms than others (see Table 5) with examples of some of these outlined below:

**Table 5:** Overview of clinical data for patients tested using the NGS panel with a clinical diagnosis of episodic ataxia type 2

ID	Age at test request (years)	Gender	Age at onset (years)	Familial history	Symptoms	Treatment response
1	2 1/2	F	2	No	Episodes of ataxia	
2	15	M	−	Yes	Episodes of ataxia associated with fever and gait ataxia and post pointing. The spell trigger by a mild head trauma	Acetazolamide, positive
3	24	M	−	−	−	−
4	37	F	−	−	Episodes of ataxia, possible hemiplegic migraine−	−
5	60	M	30	Yes	Ataxic gait, 6 episodes/year; and exercise triggers attacks	−
6	6	M	13 months	Yes (typical migraine)	Episodes of ataxia associated with fever; gait ataxia and post pointing; 4–5 attacks/year that can last for several days	No improvement with Acetazolamide
7	60	M	−	−	−	−
8	11	F	10 months	Yes	Dizzines, visual symptoms, inability to walk, nausea, occasional headache; attacks last for 30 min that can trigger with physical exertion; and sleep for 30min relieves the attacks	Acetazolamide, positive
9	4	M	−	−	−	−
10	37	F	−	−	Daily attack	−
11	26	M	−	−	−	−
12	76	F	61	Yes	Attacks of vertigo, +/‐ headache every few months	Acetazolamide, partial
13	28	M	−	Yes	Migraine, dizziness prior to ataxia attack, Charcot‐Marie‐Tooth neuropathy, pes cavus, cannot walk on heels, reflexes preserved.	Acetazolamide, positive
14	27	F	−	−	Severe ataxia, nausea, vomiting, nystagmus; four attacks/year	Acetazolamide, positive
15	66	M	childhood	Yes	Gait abnormality, no nystagmus	Acetazolamide, positive
16	42	F	−	Yes	Episodic ataxia	−
17	54	F	−	−	−	−
18	9	F	−	−	−	−
19	38	F	−	−	Vertigo, fluctuating ataxia, abnormal nerve excitability	−
20	80	F	−	−		−
21	80	M	60	No	Late onset of episodic ataxia	−
22	57	M	−	−	−	−
23	24	M	−	−	Unsteadiness, muscle myokemia, exercise induced	Very little difference with Acetazolamide
24	39	M	childhood	Yes	Vertigo, unsteadiness, rapid pulse rate, no nystagmus, gait, tone, coordination, and reflexes normal; attacks last for 2 hours and trigger by emotional stress and exertion	−
25	59	M	−	Yes	Ataxia, no headache.	−
26	18	F	10 months	No	Ataxia associated with fever, dizziness, vomiting, headache, occasional symptoms (postural hypotension, palpitations), one or two attacks/month; exercise and anxiety trigger attack; no nystagmus	Acetazolamide, positive
27	38	M	−	−	Nystagmus	−
28	37	M	−	−	−	−
29	38	M	−	Yes	−	−
30	35	M	−	Yes	−	−
31	56	F	−	No	Progressive ataxia, vertigo	−

For case 6 (a 6‐year‐old male); he presented with a clinical picture suggesting EA2. He had an episode of “encephalitis” at 13 months of age associated with marked ataxia, which resolved over 3 months. Since then, he has had 4–5 episodes of ataxia associated with fever which resolved over several days. In the last described episode, he had severe ataxia with fever, marked truncal and gait ataxia, and past pointing. He had a normal MRI result, but no response to Acetazolamide. A novel single base‐pair exchange was identified in the *CACNA1A* gene (located in intron 44) in this patient, which is predicted to affect the *CACNA1A* protein through changes to mRNA splicing sites.

In case 8 (an 11 year old female), there were episodes of ataxia since the age of 10 months, dizziness; visual symptoms; unsteadiness (inability to walk); nausea; vomiting, and occasional headaches reported during the attacks, with complete resolution of symptoms typically taking 1–2 h. Her mother (44 years of age) had similar attacks starting at an age of 5 years, improving as she got older, though pregnancies exacerbated her symptoms. During recent episodes of ataxia, she was unable to walk and suffered severe dysarthria. Both daughter and mother's symptoms were relieved by Acetazolamide. A small frameshift deletion p.Leu600 fs*41 [2 bp] was identified using the NGS panel in the index case (daughter) and confirmed by Sanger sequencing in both the index case and her mother.

In case 10 (a 37‐year‐old female), there was at least a 3 year history of episodic acute onset ataxia associated with nausea and vomiting. She also had a history of migraine and recent daily attacks were reported. A new single‐nucleotide variant (SNV) in the 3′UTR in *ATP1A2* gene was detected in this patient.

In case 13, familial periodic ataxia (paralysis) and migraine were described in both index case (a 28‐year‐old male), his brother (35 years of age) as well as their mother and all were known to respond to Acetazolamide. The brother was also diagnosed as having Charcot‐Marie‐Tooth (CMT) neuropathy; pes cavus and with inability to walk on his heels but no muscle wasting. The amino acid changing p.Asp302Asn mutation and the new synonymous SNV (Asn328Asn) in *CACNA1A* gene were both confirmed in both brothers.

In case 19 (a 38‐year‐old female), typical vertigo and abnormal nerve excitability were reported suggesting a clinical diagnosis of episodic ataxia or migraine vertigo. Although no mutation was identified in this case, a novel non amino acid changing variant was detected in the *NOTCH3* gene at the nucleotide position c.5640C>T (p.Val1880Val), which was computationally indicated to be potentially damaging.

## Discussion

Autosomal dominant (EA2) results from mutations of the *CACNA1A* gene, covering 300 kb with 47 exons. EA2 is caused by a wide range of mutations in *CACNA1A*, localized on chromosome 19p, which encodes the pore‐forming *α*_1A_ subunit of the Ca_V_2.1 Ca^2+^ channel (Ophoff et al. 1996). This subunit comprises four repeated domains (I–IV), and each domain contains six transmembrane regions (S1–S6) comprising a pore loop between S5 and S6. In our tested patients, 5 of 15 mutations detected were involved in the pore loop regions of Domains I, III, and IV of the protein. Of these, three mutations were missense mutations in Cases 9, 13, and 29, one was a truncation mutation (Case 17) and one was a previously known nonsense mutation (p.Trp1448Ter) (Case 5) (Jen et al. 2004). It is worth noting that these mutations were spread throughout the gene. This reinforces the difficulty in screening a few exons which harbor “hot spots” for mutation in the *CACNA1A* gene, an element compounded by symptomatic overlap between EA2 and other diseases.

EA2 is mainly characterized by episodes of ataxia, vertigo, and nausea lasting for minutes to hours, but a variety of overlapping clinical features with other dominant disorders like FHM1 and SCA6 have been previously described (Jodice et al. 1997; Mantuano et al. 2003; Romaniello et al. 2010), such as dysarthria, diplopia, hemiplegia, and headache (Jen et al. 2004). Attacks are triggered by emotional stress, exertion, caffeine, or alcohol in similar ways to these disorders and even common migraine. The issue with overlapping symptoms potentially confusing decisions relating to the clinical diagnosis of EA2 and treatment are exemplified in Case 18, in whom we identified a new missense mutation in the *ATP1A2* gene (p.Thr570Met), instead of *CACNA1A* where one was expected to be found. This patient suffers from highly similar symptoms to EA2 (hence this being the requested test), and all episodes of their illness were resolved by using Acetazolamide treatment. This case's result opens the door to potential clinical confusion in the precise differential diagnosis of EA2 from FHM type 2, with response to acetazolamide not being a reliable indicator of having EA2. This also leads to potential to sequence incorrect genes to identify mutations, and while this may not always lead to delays in treatment, it does have the potential to limit family planning options for patients and the ability to diagnose other family members.

Moreover, two of the known mutations presented here in two clinically diagnosed EA2 patients p.R583Q and p.Y1853* were previously described to be associated with both (FHM) and Episodic Ataxia2 (EA2). In more detail, Battistini et al. (1999) reported two sisters with p.Arg583Gln mutation who have typical hemiplegic migraine attacks associated with confusion and fever, accompanied with progressive cerebellar ataxia. In 2010, the same mutation was reported by Cleves et al. in two sisters experiencing episodic ataxia without hemiplegia and confusion (Cleves et al. 2010). Giffin et al. (2002) described a 3 year old male with FHM and ataxia symptoms linked to a nonsense mutation (p.Tyr1853Ter) in exon 37 in the *CACNA1A* gene, the attacks being unresponsive to treatment with acetazolamide. In a similar overlapping case, the mutation p.Asp302Asn (reported here in Case 13) was reported in 2014 by Jaffer et al. in a patient with episodic ataxia (Jaffer et al. [Link]), whereas the same mutation was described by Burk et al. (2014) in a German patient with dominant cerebellar ataxia and absence of recurrent ataxic episodes, see (Burk et al. 2014).

For the 16 patients for whom our panel failed to detect known or unknown mutations, we detected three previously unknown variants: in Case 6, an intronic SNV in the *CACNA1A* gene; in Case 19, a synonymous variant in the *NOTCH3* gene; and in Case 10, a SNV found in the 3′UTR of *ATP1A2*. These variants have unknown pathogeniciy, and all but Case 19′s variant were predicted to be tolerated by the* in silico* tools.

In addition, rare SNPs (<1% minor allele frequency) were also detected in 10/16 cases, some of which might be involved with a patient's individual phenotype. For instance, in Case 31 classified as having a progressive ataxia, carries the p.Arg1917Gly at c.5749C>G (rs121917956) polymorphism in the sodium channel gene. This variant is predicted to have a damaging effect when using Polyphen‐2 and Mutation Taster programs but is categorized by NCBI as a variant with an allele of uncertain significance. Moreover, this variant has been seen twice in families with febrile seizures. Wallace et al. 2001; found the variant in 1.2% of affected people, though they also found the variant in unaffected people (60 controls of unknown origin) at a similar frequency (1.7%), and therefore they considered the variant a SNP. Nevertheless, Zucca et al. (2008) reported the same variant as a causative mutation causing a severe myoclonic epilepsy of infancy (SMEI) and the variant was not present in a panel of 250 Caucasian controls. Zucca et al. also determined that the variant altered exonic splicing enhancers, potentially resulting in splice site changes in *SCN1A* (Wallace et al. 2001; Zucca et al. 2008). It is worth noting that the frequency of this SNP in a large (~600 individuals) Caucasian population from the ClinSeq project available in the NCBI SNP database is 0.3%. Additional research will be needed to determine its effect and whether it may be useful to extend the clinical phenotype spectrum of *SCN1A* mutations to include progressive ataxia along with epilepsy and familial hemiplegic migraine.

Further assessment for deleterious or disease‐causing effects using *in silico* methods for all novel variants in future might help to confirm the phenotype‐genotype correlation. In addition, family segregation analyses may be needed to confirm the contribution of these variants to the patient's phenotype. This may be especially important as such rare polymorphisms may effectively represent low penetrance mutations whose presence in disease carriers has gone unnoticed due to variable phenotype expression.

Alternatively, it is possible that these rare polymorphisms do not contribute to disease phenotype and there are mutations in other genes responsible for their illnesses. Given our discovery of a gene causing a disease with EA2 symptoms in a patient bearing an ATP1A2 mutation, this seems likely, and perhaps the use of whole‐exome or genome sequencing approaches will enhance the probability of identifying new genes and/or mutations responsible for EA2 to allow future improvement of EA2 diagnostics.

In terms of a diagnostics approach, the imprecise definitions of symptoms for the clinical diagnosis of EA2 and the large number of exons in the *CACNA1A* gene, make obtaining a clear clinical diagnosis using direct genetic testing using conventional Sanger sequencing for molecular diagnosis extremely difficult. Indeed, our detection of mutations in *ATP1A2* potentially causing EA2‐like symptoms strongly indicates that multiple gene screening could be a clinically valuable approach. Indeed, one might reasonably screen the other familial hemiplegic migraine genes FHM2 (*ATP1A2)*, FHM3 (*SCN1A)* along with FHM1 (*CACNA1A*) in case these are responsible for EA2‐like symptoms in a patient.

With the number of the mutations identified in 15 of 31 (48.4%) of the EA2 patients tested our newly developed custom panel provides an improved diagnostics tool compared to traditional exon‐by‐exon sequencing widely used in laboratories to identify the genetic aberrations of patients sharing overlapping symptoms with EA2, such as FHM and SCA6. However, these results also indicated that there may be other EA2 genes yet to be identified and to be included on future diagnostic arrays.

In comparison to panels with a very large number of genes or to whole‐exome sequencing, the small number of genes in a disease‐specific gene panel (such as our NGS multigene panel) allows a significant increase in coverage on target sequences and high read depth of all bases. This is a great help to reliably detect disease‐causing mutations, as well as to limiting the detection and validation of large numbers of unclassified variants in different genes which might not relate to the patient's phenotypes. Moreover, high read depth for all the 15 mutations identified (>200x coverage depth) (Table 3) and 100% concordance of the mutations detected by NGS panel with Sanger sequencing provides high confidence in mutation detection. This may enable elimination of Sanger sequencing to confirm the presence of the mutations if the coverage read depth is ≥100x, with Sanger confirmation perhaps reserved for less reliably detected mutations. Therefore, massively parallel sequencing of small panel genes should be considered as a screening tool to detect clear monogenic mutations of neurological disorders in which there is phenotypic and genotypic heterogeneity. Indeed, in an environment where whole exome and potentially whole genome sequencing is becoming more affordable, multi‐gene panels may become the first stage in a genes‐to‐exome‐to‐genome approach to replace the older exon‐by‐exon sequencing using Sanger methods as the clinical standard method for genetic disease diagnosis.

## Conclusions

We have developed a custom panel comprising five genes for daily routine genetic testing of (EA2). The mutational spectrum identified in this study included fifteen different mutations (6 known and 9 unknown). Among them are three small deletions (*CACNA1A* p.Thr310 fs*5 [4 bp]; Val558Serfs*13 [5 bp]; and p.Leu600 fs*41 [2 bp] which disrupt the reading frame and result in a premature stop of the CACNA1A protein at amino acid positions 314, 570, and 640, respectively, these were identified in exons 6, 13, and 14). The remaining *CACNA1A* mutations were simple amino acid substitutions. Additionally, a previously unknown mutation was identified in the *ATP1A2* gene p.Thr570Met in association with EA2 symptoms, confirming the difficulty to differentiate between the clinical features of EA2 and FHM for a precise clinical diagnosis.

On the basis of this report, we felt that transitioning to an NGS platform that performs parallel sequencing enables much more cost‐effective diagnosis and a more comprehensive diagnostics test, involving an interrogation of all implicated and related genes simultaneously. This provides the opportunity to identify novel and unexpected mutations increasing diagnostic capability. This approach reduces the difficulty for clinicians in choosing the genes to investigate due to the symptom overlap in relation to severe migraine‐related disorders, with the added consequence of keeping patient costs down.

## Conflict of Interest

The authors declare no conflict of interest.
